# Usability Evaluation of the Revised *Color Me Healthy* Symptom Assessment App: Perspectives of Children and Parents

**DOI:** 10.3390/children11101215

**Published:** 2024-10-04

**Authors:** Lauri Linder, Haley Utendorfer, Brianna Oliveros, Sydney Gilliland, Victoria L. Tiase, Roger Altizer

**Affiliations:** 1 College of Nursing, University of Utah, Salt Lake City, UT 84112, USA; victoria.tiase@utah.edu; 2 Center for Cancer and Blood Disorders Primary Children’s Hospital, Salt Lake City, UT 84113, USA; 3 School of Medicine, University of Utah, Salt Lake City, UT 84113, USA; haley.utendorfer@hsc.utah.edu; 4 College of Humanities, University of Utah, Salt Lake City, UT 84112, USA; u1286713@utah.edu; 5 Medstar Washington Hospital Center, Washington, DC 20010, USA; sgilliland@asuu.utah.edu; 6 Department of Biomedical Informatics, School of Medicine, University of Utah, Salt Lake City, UT 84108, USA; 7 Department of Population Health Science, School of Medicine, University of Utah, Salt Lake City, UT 84108, USA; roger.altizer@utah.edu

**Keywords:** childhood cancer, parents, symptoms, symptom assessment, mHealth, digital health, usability evaluation

## Abstract

Background: The *Color Me Healthy* symptom reporting app was co-designed with school-age children with cancer, their parents, and clinicians. Preliminary studies demonstrated its feasibility and acceptability; however, children and parents identified the need for additional refinements. Methods: Guided by the Technology Acceptance Model and principles of user-centered design, this study supported refinements to the *Color Me Healthy* user interface and evaluation of its usability. As the programming team completed builds of the app, school-age children with cancer and their parents participated in cognitive walkthrough usability evaluations and qualitative interviews. Usability logs documented the completion of key tasks related to reporting pain and review of child-reported data. Parents completed the Technology Acceptance Model Perceived Usefulness Scale (TAM-PUS). Interview responses were analyzed using qualitative content analysis. Results: Fourteen children (median age 8.5 years; range 6–12) and 14 parents (median age 38.5 years; range 34–49) participated in one of three usability evaluation cycles. After the third cycle, children and parents navigated the app and completed key tasks independently. Median TAM-PUS scores were 6 (range 6–8), indicating high perceived usefulness. Qualitative analyses indicated that children regarded the app as easy and fun to use. Parents emphasized the app’s developmental relevance for their child and for themselves as the child’s caregiver. Conclusions: This study demonstrates the perceived usefulness and perceived ease of use of the revised *Color Me Healthy* app. Optimizing the usability of the *Color Me Healthy* app with attention to the user needs of children and parents positions the app for wider-scale clinical implementation.

## 1. Introduction

Children with complex health conditions, including cancer, experience multiple co-occurring symptoms including pain, fatigue, nausea, vomiting, disrupted sleep, and sadness that arise from their disease as well as from the intensity of treatment necessary to achieve curation [1,2]. These symptoms interfere with children’s daily lives, negatively impact their quality of life, and may result in delays in prescribed treatment [3,4,5,6]. Consensus recommendations from the 2018 Children’s Oncology Group (COG) State of the Science Symposium: Symptom Assessment During Childhood Cancer Treatment include regular assessment emphasizing the child’s self-report and communication between the family and care team [7]. Although symptoms are recognized as the individual’s subjective experience [8], many children have difficulty providing their own verbal self-report. Elementary school-age children, specifically those 6–12 years of age, tend not to initiate reports of symptoms unless specifically asked and are often intimidated by questions from the clinical team [9]. Children may experience multiple co-occurring symptoms and can have difficulty verbally articulating the presence and associated characteristics of these symptoms. As a result, they also tend to use expressions reflecting their general feeling state such as “gross and yucky” which can be challenging for parents and clinicians to interpret [10,11].

In the United States, nearly all children have access to and regularly use mobile devices, including tablet computers, whether in the home setting for personal use or in educational settings [12]. Because children regularly interact with multiple apps, including both game-based apps designed to support learning and stand-alone games, mobile health (mHealth) apps offer child-centric solutions to support children in providing their own health reports [13,14]. In addition to their familiarity to children, mHealth apps may be a preferred alternative for providing a self-report rather than responding to multiple verbal questions. The multi-sensory experience that is offered through interacting with an app is also developmentally relevant and can foster the child’s recall, leading to a more complete and comprehensive report [13,14,15,16,17]. 

Engaging target end users in the development of mHealth apps is critical to their future adoption. For apps targeting children, this includes intentionally engaging children in their design and evaluation, giving consideration to cognitive abilities, motor skills, attention span, and emotional responses [18]. Because parents ultimately influence their child’s use of mHealth apps and supervise their use, parents must perceive a benefit to their child’s use of the app [19,20]. Therefore, engaging parents in the design and evaluation of mHealth apps for children is salient. Grounding app development efforts in a theoretical framework further allows evaluation of factors influencing future adoption.

To support symptom reporting among school-age children (6–12 years) with cancer, our interprofessional team co-designed the Android-based *Color Me Healthy* app with school-age children with cancer, their parents, and clinicians [21]. We intentionally chose a tablet computer for delivering the app because of its familiarity. We also sought to accommodate the developing fine motor skills of children as young as 6 years of age as well as potential neurosensory deficits that children may experience because of their disease or as a treatment-related side effect [22].

The *Color Me Healthy* app features a customizable avatar and interactive home page to guide children through the app. The symptom tracking feature includes eleven common cancer-related symptoms derived from child versions of the Memorial Symptom Assessment Scale (MSAS) [23,24]. Children can localize additional site-specific symptoms, including mouth sores, bruising, and numbness/tingling to areas of the body including the head, mouth, chest, abdomen, and extremities using a drawing feature. Other app features include a daily goal page, a diary, and a sketch pad. Goals include common daily expectations for school-age children: taking a bath, brushing their teeth, a physical activity goal, and a learning/creative activity goal.

Our initial trial of the *Color Me Healthy* app demonstrated its feasibility and acceptability among children receiving cancer treatment and their parents [25]. We also demonstrated its ability to support children not just to communicate their symptoms, but to allow parents and clinicians to gain insights into their daily life experiences [19,26]. Feedback from children and parents, however, identified the need for additional refinements to the app prior to large-scale implementation and clinical evaluation of its efficacy. These included additions to the user interface to support ease of navigation, minimizing the potential for missing data, and enhancing user engagement through incentives [25].

### 1.1. Theoretical Framework

We used the Technology Acceptance Model (TAM) as the framework for this study [27,28,29]. The TAM posits that key determinants of the actual use of a given technology include its perceived ease of use and perceived usefulness. Perceived ease of use is the individual’s perception that using a given technology will be free of effort. Perceived usefulness is the extent to which an individual believes that using a given technology will enhance their job or task performance. The overall design of the technology as well as user engagement in development and implementation of the technology further determine actual use [27]. 

The overall project was further guided by principles of user-centered design (UCD) which emphasizes (1) design based on an understanding of the users, tasks, and environment; (2) involvement of users through design and development; (3) design driven by user-centered evaluation; (4) iterative process; (5) the whole user experience; and (6) multidisciplinary skills and experiences [30,31].

### 1.2. Purpose

The purpose of this study was to revise the *Color Me Healthy* app and evaluate the usability of the revised app with attention to ease of use and perceived usefulness from perspectives of target users, school-age children with cancer and their parents. By observing target users’ efforts to complete key tasks within the app, we sought to identify and address any problems or areas of confusion in the user interface.

## 2. Materials and Methods

### 2.1. Design

This iterative usability study evaluated the usability of revisions to the *Color Me Healthy* symptom assessment app by school-age children with cancer and their parents with attention to ease of use and perceived usefulness. A usability study specifically seeks to determine whether target users can complete a set of realistic tasks relevant to the technology, typically through observation [32,33]. Usability testing is iterative and is repeated using new sets of target users as needed. Results of usability tests guide developers and designers in making refinements to the given technology. Refinements may include changes to the design, interface, or functionality to optimize ease of use for the target user. This study included iterative cycles of completing refinements to the *Color Me Healthy* user interface followed by in-person, moderated cognitive walkthrough observational evaluations and qualitative interviews with children receiving cancer treatment and their parents.

### 2.2. Study Setting

The work of revising the *Color Me Healthy* app was completed by the Therapeutic Games and Apps Lab (The GApp Lab) at the University of Utah. The GApp Lab is a collaboration between the University of Utah’s Entertainment Arts and Engineering department and Center for Medical Innovation and brings together graduate students and faculty that leverage game design to create clinically relevant innovative mHealth solutions [34]. 

Child and parent study participants who evaluated the revised app were recruited from the ambulatory oncology clinic of the Center for Cancer and Blood Disorders (CCBD) at Primary Children’s Hospital in Salt Lake City, Utah. Primary Children’s is a Children’s Oncology Group-affiliated, free-standing children’s hospital serving the multi-state region of the Intermountain West of the United States. The CCBD includes a 32-bed inpatient unit and an ambulatory clinic with a daily census of over 45 children and adolescents.

### 2.3. Study Sample

Consistent with the purpose of a usability study, the study recruited individuals representing target users for the *Color Me Healthy* app. Inclusion criteria for child participants were to be between 6 and 12 years of age, currently receiving treatment for cancer, and having completed at least one cycle of cancer treatment. Eligible parents were the parent or legal guardian of a child participant. Additional inclusion criteria for children and parents included the ability to speak and understand English and be physically and cognitively able to complete study procedures. Children who had completed therapy and their parents were excluded.

Guided by usability testing theory which posits that 5 or 6 users will identify nearly 85% of usability problems [35], we sought to accrue at least 5 child/parent dyads to evaluate each new build. The goal for each cycle was to include at least 2 boys and 2 girls with at least 2 younger (6–8 years) and 2 older (9–12 years) to support gender and age group representation. We also sought to recruit at least one child from a racial/ethnic minority group in each cycle. We also sought to preferentially recruit fathers, when feasible.

### 2.4. Ethical Considerations

Institutional review board approval for the study was granted by the University of Utah (IRB_00133421). Our team received a waiver of authorization to conduct electronic health record screens of patients scheduled for ambulatory oncology clinic visits or inpatient admissions to the oncology unit to identify children and parents meeting eligibility criteria. Those identified as meeting initial criteria were reviewed with the clinical team to confirm eligibility. Eligible families were informed of the opportunity to participate in the study by a member of their clinical team. Families who agreed to be contacted were then approached by a member of the study team who further explained the study procedures. Parents provided written permission for the child’s participation along with their own informed consent. All children provided verbal assent. Children 7 years of age and older provided written assent.

### 2.5. Cognitive Walkthrough as a Usability Evaluation Method

Cognitive walkthrough is a task-based usability evaluation that evaluates the learnability of a given system from the perspective of a new user [36]. This method is well suited to new users and usability evaluation of working prototypes. Key objectives of a cognitive walkthrough include determining whether the user will (1) attempt to achieve the correct result, (2) notice that the correct action is available, (3) associate the correction action with the result they seek to achieve, and (4) see that progress is made toward the goal. Although the cognitive walkthrough method was initially developed for use with experts rather than actual system users, the method has been successfully adapted for use with actual target end users [37]. In-person, moderated cognitive walkthrough evaluations supported direct observation of users’ actions and opportunities for users to verbalize their thoughts while navigating the app, providing additional real-time insights into how they interpreted the user interface [38].

### 2.6. Study Measures

Measures in the study were selected to evaluate constructs with the Technology Acceptance Model [27]. Table 1 summarizes Technology Acceptance Model constructs evaluated in this study and the data source(s) used for evaluation.

#### 2.6.1. Usability Logs

Because the focus of the usability evaluations for this project related to the learnability of the revised interface, we created usability logs for children and parents to assess each user’s ability to complete key intra-app tasks based on their respective roles. First, we created task lists, one for child users and one for parent users, to complete during cognitive walkthrough evaluations. Task lists emphasized each user’s respective role related to the revised symptom assessment feature. Tasks specifically addressed the documentation of pain and its associated characteristics, including localization to a given area of the body. Because of the high prevalence of pain among children with cancer [2], we anticipated that most children would be able to recall a current or recent pain experience to report. Key tasks for child users included (1) accessing the symptom assessment feature, (2) reporting pain and its associated attributes as a general symptom, (3) localizing pain to a specific area of the body, and (4) reviewing their symptom report in the app’s history feature. Key tasks for parent users included (1) accessing the history feature, (2) reviewing a daily symptom summary, and (3) accessing a longitudinal symptom report. Tasks were scored as “completed independently”, “required verbal prompt”, or “required physical prompt”. Usability logs also included a notes section for each task for the observer to indicate any additional details. 

#### 2.6.2. Qualitative Interviews

Qualitative interviews for children and parents included questions that have been used in prior studies evaluating the acceptability of mHealth software [25,39]:

- What did you like the most about the app?
- What did you like the least about the app?
- What should we change about the app to make it better?

An additional interview item solicited the child and parent’s preference for having the child report symptoms using an app or paper-based tool and associated rationale.

#### 2.6.3. Technology Acceptance Model Perceived Usefulness Scale (TAM-PUS)

The 4-item TAM-PUS rates aspects of perceived usefulness on a 7-point Likert scale with responses ranging from 1 = “highly likely” to 7 = “highly unlikely” [40]. Responses are summed with lower scores indicating greater perceived usefulness. Acceptable internal consistency (α = 0.87–0.98) has been reported [40], and the scale can be completed in less than 5 min.

### 2.7. Procedure

#### 2.7.1. Revising the *Color Me Healthy* App

The work of revising the *Color Me Healthy* app involved principles of Agile software development, which emphasize collaboration and the frequent delivery of working software [41]. The project began with a Design Box meeting in January 2021. The Design Box method is an inductive process that considers the needs of end users from the beginning [42,43]. The Design Box also considers the technology, aesthetics, audience, and underlying question prior to proposing a design. Specific objectives for app revisions, guided by our prior work, were to ensure improved intra-app navigation to ensure consistency across screens, expand the app’s content for localizing symptoms, enhance data visualization, and increase intra-app user incentives and interactions. Programming was completed by graduate students enrolled in the University of Utah’s Entertainment Arts and Engineering (EAE) program. Students worked in the roles of producer, artist, and engineer based on their area of specialization in the EAE program. 

In addition to addressing initial priorities identified through the Design Box meeting, students made iterative revisions based on feedback from child and parent users identified during usability evaluation cycles. Changes were made at the conclusion of each cycle prior to starting the next.

#### 2.7.2. Cognitive Walkthrough Evaluations and Interviews

Consistent with the purpose of a usability study, child and parent participants were asked to complete a specified set of real-world tasks relevant to the *Color Me Healthy* app. These were completed using moderated, in-person cognitive walkthrough evaluations which allowed an observer to note areas of confusion or difficulty.

Cognitive walkthrough evaluations and interviews were completed between August 2021 and March 2022 during scheduled visits in the ambulatory oncology clinic to minimize participant burden related to time and travel and to support the accrual of a study sample representative of the larger clinical catchment area. All sessions were audio recorded.

Cycles of moderated user-centered cognitive walkthrough usability evaluations and qualitative interviews were conducted as new versions (builds) of the app were available. Cycles were repeated until complete saturation was reached: revisions to the user interface had been completed, all identified bugs in the code had been addressed, and users were able to navigate the user interface without requiring physical prompts to complete tasks within the app. 

During each cognitive walkthrough evaluation, a study team member completed a usability log to document participants’ ability to complete key tasks, document potential errors (“bugs”) in the app’s code and describe any difficulties in completing key tasks. In addition to completing the requested tasks set forth in the usability logs, both children and parents had the option to interact with other features within the app.

After completing the cognitive walkthrough evaluation, children and parents participated in qualitative interviews. Interviews supported additional feedback which, combined with the observational data, helped guide refinements to the user interface. Parents also completed the 4-item TAM-PUS. Each child and parent participant received a gift card as a token of appreciation following their completion of study procedures.

### 2.8. Data Management and Analysis

Data management and analyses were guided by key constructs within the TAM (Table 1). Quantitative data from usability logs and TAM-PUS scores were entered into REDCap [44]. Frequencies of usability log responses (i.e., completed independently, required verbal prompt, required physical prompt) were reviewed after each cycle to prioritize subsequent refinements to the user interface. Although the study was not powered to detect statistically significant differences in TAM-PUS scores, individual item and summed TAM-PUS scores were also reviewed following each cycle to examine trends in scores and to identify priorities for subsequent refinements. 

Audio recordings of evaluations and interviews were transcribed verbatim to support qualitative analyses. Transcripts were also reviewed for feedback to guide programming refinements. Summaries of quantitative and qualitative data were also shared with the programming team following each evaluation cycle to guide additional refinements.

Qualitative data were further analyzed using qualitative content analysis [45,46,47]. Transcripts were anonymized and uploaded into Dedoose for analysis [48]. Three study team members (LL, HU, and BO) reviewed and coded interview transcripts. Study team members reviewed each transcript twice to gain a larger sense of the entirety of the data prior to identifying units of meaning and assigning codes. Units of meaning were defined as several words or a statement(s) that aligned with TAM construct definitions. Each team member reviewed and coded transcripts independently. The three team members then met together to review responses and to reach consensus.

Coding was conducted in a two-phased approach and included both deductive and inductive coding processes [47]. First, an a priori coding schema was used to identify units of meaning aligning with constructs within the TAM. Units of meaning were further delineated to identify statements made by child and parent participants. Second, an inductive open coding process was used to identify categories and subcategories within each grouping of excerpts based on each TAM construct. Frequencies of categories and subcategories were then summarized and compared across TAM constructs.

## 3. Results

### 3.1. Participants

Fifteen child/parent dyads enrolled in the study, and fourteen completed study procedures (Table 2), each participating in one of three usability evaluation cycles. One dyad was unable to complete study procedures due to a lack of time during the clinic visit following enrollment and the subsequent lack of an opportunity to schedule time to complete procedures. Another family declined participation after being approached by the study team.

### 3.2. Summary of Revisions and Refinements to the Color Me Healthy App

Initial revisions emphasized the user interface with attention to updating the art and color scheme, improving intra-app navigation, and refining symptom reporting and data visualization (Table 3).

**Figure 1:**
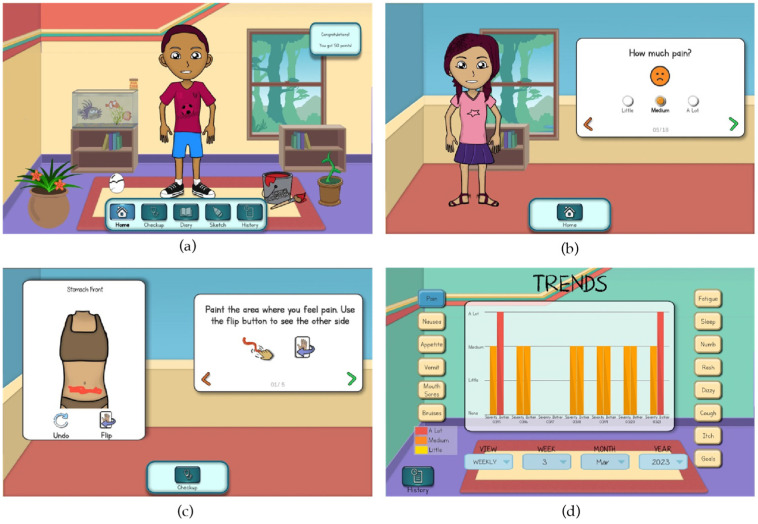
(**a**) Revised home screen; (**b**) revised screen for rating general symptoms; (**c**) revised screen for localizing symptoms; (**d**) revised screen for reviewing trends in symptom characteristics.

Subsequent refinements were guided by child and parent user feedback from cognitive walkthrough cycles (Table 4). Key refinements following Cycle 1 emphasized adjustments to the history feature to support the interpretation of child-entered data as well as revisions to the avatar skin tone options. Key refinements following Cycle 2 emphasized the child user experience by increasing interactivity with features on the home screen such as animations for the fish tank. The response option to the first item in the daily checkup, “How are you feeling today?”, was changed to emojis rather than free text to create a more supportive initial impression for children with limited reading skills. The history features were further refined to support interaction between the daily and longitudinal reports to further facilitate interpretation of child-reported data. Figure 1a–d illustrate key refinements to the user interface. All screens are accessible to child and parent users.

### 3.3. Quantitative Results

Cognitive walkthrough evaluations ranged from 10:39 to 29:30 min (median 18:28) for children and 4:52 to 17:31 min (median 6:23) for parents.

#### 3.3.1. Perceived Ease of Use: Objective Usability

Table 5 and Table 6 summarize child and parent participants’ ability to navigate the revised *Color Me Healthy* app to complete key tasks related to reporting pain as a general and localized symptom (child participants) and then to access and interpret child-entered data (child and parent participants). More detailed summaries are provided in Supplementary Tables S1 and S2.

Collectively, children accessed the general symptom feature and reported pain as a general symptom easily. All child participants in Cycle 1, however, required at least one verbal prompt with two requiring physical prompts to complete requested tasks. With subsequent builds, tasks associated with localizing symptoms and reviewing the history page became more learnable as the programming team made additional refinements based on user feedback.

Developmental abilities may have influenced objective usability across cycles. Two of the four participants who were six years old (one each in Cycles 1 and 2) required initial verbal prompts with each aspect of localizing pain; however, each was subsequently able to initiate localizing pain to other areas of the body. Of these two children, one required physical prompts to review the history page; for the other, this aspect of the usability evaluation was deferred. Two other children who were six years of age completed all requested tasks within the cognitive walkthrough evaluation independently.

Most parents, likewise, completed key tasks within the revised *Color Me Healthy* app easily, providing evidence of learnability of the app’s revised user interface. One parent in Cycle 1 required a verbal prompt to view the daily report of pain that had been localized to an area of the body. This was addressed prior to the start of Cycle 2 with the addition of a symbol on areas of the avatar’s body to which one or more symptoms had been localized.

#### 3.3.2. Perceived Usefulness

Thirteen parents completed the TAM PUS. The median summed score across all three cycles was 6 (range 6–8). Median scores for individual items ranged from 1 to 2, indicating that parent participants across all cycles perceived the app as “extremely” to “quite” likely to be useful to support them in recognizing and responding to their child’s pain.

### 3.4. Qualitative Results

We mapped 227 units of meaning from child participants and 187 units of meaning from parent participants to TAM constructs for inclusion in the qualitative analyses. Table 7 summarizes the frequency with which constructs were represented among the excerpts along with subcategories and exemplar statements.

#### 3.4.1. Perceived Ease of Use: Computer Self-Efficacy

Computer self-efficacy reflects the degree to which individuals believe that they can perform the given task using the computer [49,50]. Qualitative data from child and parent participants complemented observations documented on usability logs, indicating that both children and parents could complete key tasks and found the revised *Color Me Healthy* app easy to use. Statements made by children and parents while completing cognitive walkthrough evaluations, such as “*there we go*”, indicated that they could navigate the user interface correctly when requested by the investigator to complete key tasks (mother of a 10-year-old boy with lymphoma). Comments by children and parents, such as “*Uh, where this is?*” (10-year-old boy with lymphoma) or “*For the trends uhhh oh sorry that was not it that was right there and … help, I hit it again*” (mother of 9-year-old girl with leukemia), provided further detail regarding occasions in which they required a verbal or physical prompt to complete a task or reflected suggestions to improve the intuitiveness of the user interface.

Six children also made comments while initiating interactions with features of the app in addition to the tasks requested as part of the cognitive walkthrough. Comments reflected children’s confidence and familiarity in navigating software: “*I want to push this one*” (9-year-old boy with lymphoma) or “*I’m gonna try that*” (8-year-old girl with leukemia).

Parents’ comments also reflected their perception of their child’s computer self-efficacy. The father of a 9-year-old boy with leukemia commented, “*um it seems like kids are pretty intuitive when it comes to apps*”. Additionally, comments from two parents of 6-year-old children addressed their child’s current reading level as potentially influencing their child’s self-efficacy in using the app: “*For someone his age, maybe little images are easier to [use as response options] rather than having to type out words. Now he wouldn’t be able to just type out words on his own*” (mother of a 6-year-old boy with leukemia).

#### 3.4.2. Perceived Ease of Use: Computer Playfulness 

Computer playfulness reflects the intrinsic motivation or degree of cognitive spontaneity in the computer-related interactions [27]. Statements and reactions from both children and parents during the cognitive walkthrough evaluations reflected aspects of spontaneity and reciprocity as they interacted with the app’s features. Parents and children also made statements that reflected a general, overall sense of the app’s playfulness: “*it was very interactive*” (12-year-old boy with leukemia) and “*it’s fun*” (mother of 8-year-old girl with leukemia). Three children, while designing their avatar, made spontaneous comments while selecting features that represented themselves, including hair, clothing, and color options: “*Ooh! It’s like me*” [in response to selecting the hair option designed to reflect scraggly hair that was growing back] (10-year-old boy with lymphoma). 

Both children and parents provided suggestions to increase the playfulness of the app with attention to increasing interactions with the app’s features and earning rewards. Suggestions included ideas for additional features such as owning and caring for a pet or enhancements to existing features such as adding animations to the fish tank on the home page. These suggestions were prioritized and incorporated based on frequency of requests as well as the time and resources necessary to complete these features. Table 3 and Table 4 list additional interactive features that were implemented. 

#### 3.4.3. Perceived Ease of Use: Perceived Enjoyment

Perceived enjoyment reflects the degree to which using the system is perceived to be enjoyable in and of itself [51,52]. Statements from both children and parents reflected enjoyment of the app in general: “*All of it*” (11-year-old girl with leukemia) and “*I think it’s great*” (mother of a 6-year-old boy with leukemia). Children and parents also related specific features that they found enjoyable to use. Children, in particular, endorsed interactions with the drawing feature as enjoyable (“*the drawing part*” (10-year-old boy with leukemia)) as did their parents: “*Oh cute. That’s so cute*” (mother of an 8-year-old girl with leukemia).

#### 3.4.4. Perceived Usefulness: Job Relevance

Job relevance is a cognitive process, specifically, the degree to which individuals believe that the system is applicable to their job [40]. In the context of this project, “job” was conceptualized as the child and parent’s respective roles in reporting and responding to symptoms. Children’s statements were primarily present-oriented as they interacted with the app’s features to generate a symptom report: “*My stomach was just like hurting really*” (9-year-old girl with leukemia) and “*It’s itchy and it hurts in the same spot*” (6-year-old boy with leukemia). Parents spoke to the app’s potential to support their recall of the child’s symptoms in relation to the child’s treatment plan—“*Then you can see how you’re feeling on different chemos, too. That’s pretty awesome*” (mother of 10-year-old boy with leukemia)—as well as helping them better understand their child’s experience: “*I like how you’re taking a record of how they’re feeling and letting them express the way they are feeling better. They can explain a little bit better and go into more detail*” (father of a 9-year-old boy with leukemia).

Children also related that reporting symptoms using an app was physically easier than using a paper-and-pencil tool (“*Just easiest. And I don’t really have to write*” (10-year-old boy with leukemia)), easier than providing a verbal report, and helped with recall, “*Cause I might forget other things that I did*” (8-year-old girl with leukemia).

Parents spoke of the developmental relevance of the app in relation to their child’s confidence in providing a verbal report and that the app could support documentation of detail that the child might not otherwise verbalize: “*…that would just be so much easier for him and he’d probably be more honest because he wouldn’t have to write as much so it’s just clicking*” (father of a 9-year-old boy with leukemia) and “*[she] doesn’t like being asked questions whenever she’s in a lot of pain and they’re constantly asking her where like, this that. Like she freaks out so this will just make it easier that if we just got this then she can localize it and show where it’s actually hurting without like because in her mind she can process where it is and do it on here and then if when people ask her questions because when you’re in pain the disassociation happens to where she doesn’t know how to answer when you’re asking her so I think it I just think it would be a lot better.*” (mother of 9-year-old girl with leukemia).

Parents endorsed a tablet computer as relatable and familiar technology that children were already using for games and schoolwork. The tablet was also regarded as a relevant tool for reporting in the context of treatment-related peripheral neuropathy which had impacted the child’s fine motor skills.

#### 3.4.5. Perceived Usefulness: Result Demonstrability 

Result demonstrability refers to the extent to which the user believes that the results of using the system can be observed and communicated [27]. Statements from both children and parents reflected the general endorsement and functional capacity of the daily and longitudinal history features, e.g., “*Oh that’s really cool it was actually tracking it that’s awesome*” (12-year-old boy with leukemia) and “*seeing and being able to know when things were going on. I like that*” (mother of 10-year-old boy with lymphoma). Children and parents also accurately interpreted data within these features: “*Numb/tingly, yeah, … and then little sores and pain*” (mother of 10-year-old boy with lymphoma).

Several parents made suggestions for improving the interpretability of data within the history feature such as adjusting font size, the location of labels and symbols within reports, and terms for reverse regions of the body. Each of these recommendations was incorporated.

## 4. Discussion

Effective symptom management is essential to the quality of life of children undergoing cancer treatment. Symptom management begins with assessment and requires developmentally relevant approaches to elicit the child’s self-report and then facilitate communication with parents and clinicians who can act on the child’s behalf [7]. Digital health tools, including apps, are proposed to be of clinical utility to support symptom tracking for individuals with cancer and other chronic illnesses between clinical visits [53,54,55]. Additionally, the Office of the National Coordinator has prioritized the use of patient-generated health data, such as that from patient-facing apps, in routine clinical care [56]. To support future adoption of patient-facing mHealth apps in clinical care, their development, evaluation, and subsequent revisions should be theoretically based. 

This study provided evidence of the ease of use, including the learnability, of the revised *Color Me Healthy* app as users completed requested intra-app tasks and initiated interactions with other app features. The project also builds on our prior work of engaging children and parents in the evaluation of prototypes and intentionally incorporating features of relevance to them. Children and parents completed key tasks related to reporting pain and reviewing pain reports easily, with both groups requiring fewer prompts following the revisions completed after the first cycle of cognitive walkthrough evaluations. Most child participants also initiated interactions with other app features independent of the interviewer’s guidance, specifically the drawing feature as well as opportunities to further personalize the avatar.

Because children’s developmental abilities can vary, designing technology that can capture the abilities and interests of children within a given developmental stage can be challenging. School-age children remain largely present-oriented and concrete thinkers [57]; therefore, providing a resource that is perceived as fun and easy to use is a priority. Of note, children’s suggestions for improvement largely related to opportunities for increasing intra-app engagement and gamification such as interacting with the fish on the home screen and earning points for completing key tasks. Attention to computer playfulness as an aspect of ease of use [27] further influences the perceived usefulness of the technology and invites future interaction by potentially decreasing potential burden associated with the task of symptom reporting. 

Our recruitment goals allowed us to accrue a study sample was evenly divided between younger (ages 6–8 years) and older (9–12 years) school-age children. This allowed us to identify and address potential age-based differences related to the usability of the app. Because most digital health interventions for children with cancer that involve self-reporting symptoms have targeted children who are 8 years of age through to adolescents [20,58,59,60], we sought to be intentional in addressing usability among younger school-age children who may not yet be reading independently and have developing fine motor skills. Although reading abilities varied among the four participants who were six years of age, each independently completed intra-app symptom reporting tasks most relevant for child users, specifically rating the severity and distress associated with symptoms and localizing symptoms to specific areas of the body. Based on observations and feedback from child and parent participants, we adjusted the question “How are you feeling today?” from a free text response to an emoji-based response item. Comments from older children reflected maturing developmental abilities, relating what they were reporting during the usability evaluation to their own personal experience and reflecting on seeing their own data reflected in daily and longitudinal reports.

Uptake of digital health interventions for children also requires engagement of the child’s parents or caregivers. Parents are key to supporting the child’s adherence with requested symptom reporting [19,20,25]; therefore, they must perceive interventions as beneficial to their child. As the ones who interpret and respond to the child’s reported symptoms, whether through initiating interventions at home or facilitating communication with the clinical team [7,19], parents must also perceive digital health interventions as relevant to their roles. Within this study, parents’ comments indicated the perceived relevance of the app to their caregiving role in monitoring and interpreting trends in their child’s symptoms. Additionally, parents perceived the app as of value to their children, speaking of the developmental relevance of engaging children in providing their own symptom report and regarding the tablet computer-delivered app as relatable technology.

### 4.1. Directions for Future Research

Future directions include theory-based evaluation of the efficacy of the revised *Color Me Healthy* app to support symptom management and its implementation in clinical settings. Frameworks such as the Obesity-Related Behavioral Intervention Trials (ORBIT) Model [61,62] and the National Institutes of Health (NIH) Stage Model for Behavioral Intervention Development [63] provide a structure for developing and refining digital health interventions prior to evaluation in clinical trials. Frameworks such as the Consolidated Framework for Implementation Research (CFIR) [64] and the Strategic Implementation Framework [65,66] provide a basis for evaluating the implementation of digital health interventions within health systems.

Outcomes of interest for future studies include those involving children, parents, clinicians, and health systems. Prior studies have demonstrated the feasibility of longitudinally collecting electronically reported symptom-related data from children and adolescents as well as preliminary efficacy to alleviate symptoms [20,24,58,59]. Further work is needed to establish the efficacy of mHealth interventions to manage children’s symptoms as well as the optimal frequency for symptom reporting across the cancer care trajectory [7]. Outcomes of interest for children could include a reduction in the number of days with moderate or severe symptoms [67] and/or improved quality of life.

Because parents have the responsibility to recognize and respond to children’s symptoms in the ambulatory setting, research should consider the added time and effort for parents. Prior studies have indicated that perceived added burden for parents may influence the child’s adherence to mHealth interventions [20,25]. Additional outcomes of interest of relevance to the family include perceived quality of communication with the clinical team regarding the child’s symptoms and cost savings, with attention to both direct and indirect costs, associated with the child’s cancer care.

Effective symptom management also requires engagement of the clinical team [7]; therefore, research should be intentional in addressing the clinician experience. This includes clinician input for integrating child-reported data into the clinical workflow, including accessibility of data within EHR systems and having data presented in a manner that is actionable. Additional outcomes of interest include clinicians’ perceptions of time saved through the inclusion of children’s app-generated symptom reports.

Achieving the goals of the Office of the National Coordinator for Health Information Technology (ONC) to increase the routine use of patient-generated health data requires an infrastructure that supports interoperability of patient-owned devices with EHR systems [68]. Future development of *Color Me Healthy* using the SMART^®^ on FHIR specification provides a scalable, standards-based approach for data exchange that can be implemented across EHR systems and healthcare organizations [69,70,71,72]. Further infrastructure development could include the development of algorithms to provide alerts to the clinical team as well as the integration of care process models to guide clinical care in response to children’s self-reported data. Such efforts could position health systems to monitor adherence to guideline-concordant care and to evaluate potential cost savings associated with unplanned clinical encounters that were averted through earlier intervention.

Although the *Color Me Healthy* app was initially developed and evaluated among children with cancer, the symptoms that are assessed within the app are not unique to cancer alone. As such, the app has the potential for broad-scale implementation to support symptom management among children with other acute and chronic health conditions and for comparing symptom outcomes across institutions and among populations within institutions. Additional directions include creating other language versions of the app (e.g., Spanish and French) with attention to cultural relevance to support its accessibility to a more diverse target audience.

### 4.2. Limitations

Limitations of this study include recruitment at a single medical academic center. The racial and ethnic diversity of the study sample was limited due to the criterion for child and parent participants to be able to speak and understand English. Although the sample size was small, the sample size aligned with the overall scope and objectives of usability testing. Child participants were primarily diagnosed with leukemia which limited input from children and parents representing other diagnostic groups. The symptoms represented in the *Color Me Healthy* app, however, are ones that are common across diagnostic groups [1,2,3,4,5]. Additionally, *Color Me Healthy* is targeted toward elementary school-age children (typically, ages 6–12 years), and we recruited a sample that was evenly divided between younger (ages 6–8 years) and older (9–12 years) age groups, providing age-based representation. Although parent participants were primarily mothers, each evaluation cycle included one father.

## 5. Conclusions

This study demonstrated the usability of the revised *Color Me Healthy* app among school-age children with cancer and their parents. Iterative usability testing supported refinements that were responsive to child and parent feedback. Child and parent perspectives of the app’s perceived ease of use and perceived usefulness aligned with constructs in the TAM and reflected their respective roles as potential end users. Optimizing the usability of the *Color Me Healthy* app through usability testing with attention to the user needs of children and parents positions the app for wider-scale clinical implementation to support symptom management. 

## Figures and Tables

**Table 1:** TAM constructs and data source(s).

Determinants of Perceived Usefulness	
Determinant	Data Source(s)
Job relevance	Qualitative interviews and TAM Perceived Usefulness Scale (PUS)
Output quality	Qualitative interviews and TAM-PUS
Result demonstrability	Qualitative interviews and TAM-PUS
Perceived ease of use	Usability logs and qualitative interviews
**Determinants of Perceived Ease of Use**	
**Determinant**	**Data Source**
Computer self-efficacy	Qualitative interviews
Computer anxiety	Qualitative interviews
Computer playfulness	Qualitative interviews
Perceived enjoyment	Qualitative interviews
Objective usability	Usability logs

**Table 2:** Participant characteristics.

		Cycle 1	Cycle 2	Cycle 3	Total
Child participants					
Gender					
	Male	3	3	3	9
	Female	2	2	1	5
Age					
	6–8 years	2	2	3	7
	9–12 years	3	3	1	7
	Median (range)	9 (6–10)	10 (6–12)	8 (6–9)	8.5 (6–12)
Ethnicity					
	Hispanic/Latino	0	2	2	4
	Not Hispanic/Latino	5	3	2	10
Race					
	African American	0	0	1	1
	White	5	3	3	11
	More than one race	0	2	0	2
Diagnosis					
	Acute lymphoblastic leukemia	5	4	4	13
	Non-Hodgkin lymphoma	0	1	0	1
Months since diagnosis					
	Median (range)	11 (2–15)	10 (6–16)	2.5 (2–24)	10 (2–24)
Parent participants					
Gender					
	Male	1	1	1	3
	Female	4	4	3	11
Age in years					
	Median (range)	42 (34–46)	37 (34–44)	38.5 (37–49)	38.5 (34–49)
Ethnicity					
	Hispanic/Latino	0	2	1	3
	Not Hispanic/Latino	5	3	3	11
Race					
	White	5	4	3	12
	More than one race	0	1	1	2

**Table 3:** Initial revisions to the *Color Me Healthy* app.

Art and Color Scheme: Updating the color scheme and creating new art to support expanded app features
Revised color scheme for the home page and app features; Updated art for the avatar with new clothing options and color palette for customization; Revised art for areas of the body including posterior (reverse) to better localize symptoms.
**Intra-app Navigation:** **Supporting user navigation within and across app features**
Buttons to navigate to specific features within the app now located at the bottom of the home screen; Added a button specific to the symptom assessment feature, designated as “Check Up”; Addition of a “save” feature before navigating away from the symptom assessment feature to avoid missing data.
**Symptom Reporting and Data Visualization:** **Improving processes for reporting symptoms and visualizing daily and longitudinal data**
Inclusion of radio buttons (rather than slider bars) to rate symptom severity and associated bother; Inclusion of emojis with associated colors (green/yellow/orange/red) to align with symptom severity and bother ratings; Ability to localize symptoms to anterior (front) and posterior (reverse) regions of the body; Individual colors to localize specific symptoms; Initial drafts of daily and longitudinal reports.

**Table 4:** Summary of refinements to the *Color Me Healthy* app guided by cognitive walkthrough evaluations.

Refinements Completed Following Cycle 1	
	Revised skin tone color options for the avatar to include four options rather than three with the default not being the lightest option; Selected skin tone was retained within the app, e.g., art assets that support localizing symptoms to specific regions of the body retain the selected skin tone of the avatar; Revised labels on reverse areas of the body to reflect anatomic location more accurately (e.g., “upper back” rather than “chest” when chest was reversed); Added symbols to indicate regions of the body to which symptoms had been localized when reviewing the daily symptom summary; Adjusted the axes (scale and labels) and increased font size for viewing longitudinal trends on the history page; Adjusted sensitivity on the history page when transitioning from the daily summary to the longitudinal trends page.
**Refinements Completed Following Cycle 2**	
	Revised the daily check-up item, “How are you feeling today?”, to appear as the first item and to feature emojis as response options rather than a free text response; Added animations to the fish tank on the home page and the ability to feed the fish; Added an egg that hatches into a pet to the home page; Added sound effects for actions within the app; Adjusted initial music so that it stops playing after the person logs in; Addition of badges to recognize completion of key tasks for 5 and 10 days; Adjusted the location of symbols on the avatar to indicate localized symptoms; Implemented interaction between the daily and longitudinal symptom reports; Adjusted width of scroll bars on intra-app features.

**Table 5:** Summary of child participant cognitive walkthrough evaluations.

		Completed All Tasks Independently *n* (%)	Required One or More Verbal Prompts *n* (%)	Required One or More Physical Prompts *n* (%)
Cycle 1 (*n* = 5)				
	Report pain as a general symptom	5 (100)	0 (0)	0 (0)
	Localize pain to an area of the body	1 (20)	4 (80)	0 (0)
	Review report in the history	2 (40)	1 (20)	2 (40)
Cycle 2 (*n* = 5)				
	Report pain as a general symptom	4 (80)	1 (20)	0 (0)
	Localize pain to an area of the body	4 (80)	1 (20)	0 (0)
	Review report in the history ^1^	4 (80)	---	---
Cycle 3 (*n* = 4)				
	Report pain as a general symptom	4 (100)	0 (0)	0 (0)
	Localize pain to an area of the body	3 (75)	1 (25)	0 (0)
	Review report in the history	4 (100)	0 (0)	0 (0)

^1^ This aspect of the walkthrough evaluation was deferred for one participant in Cycle 2.

**Table 6:** Summary of parent participant cognitive walkthrough evaluations.

		Completed All Tasks Independently *n* (%)	Required One or More Verbal Prompts *n* (%)	Required One or More Physical Prompts *n* (%)
Cycle 1 (*n* = 5)				
	Access daily symptom report	5 (100)	0 (0)	0 (0)
	Access localized symptom report	4 (80)	1 (20)	0 (0)
	Access longitudinal symptom report	4 (80)	1 (20)	0 (0)
Cycle 2 (*n* = 5)				
	Access daily symptom report	5 (100)	0 (0)	0 (0)
	Access localized symptom report	5 (100)	0 (0)	0 (0)
	Access longitudinal symptom report	5 (100)	0 (0)	0 (0)
Cycle 3 (*n* = 4)				
	Access daily symptom report	4 (100)	0 (0)	0 (0)
	Access localized symptom report	4 (100)	0 (0)	0 (0)
	Access longitudinal symptom report	4 (100)	0 (0)	0 (0)

**Table 7:** Technology Acceptance Model constructs, subcategories, and exemplar statements by children and parents.

Child Participants		Parent Participants	
Construct and Associated Subcategories	Exemplar Statements	Construct and Associated Subcategories	Exemplar Statements
Perceived Ease of Use			
Computer self-efficacy	(76 statements by 13 children)	Computer self-efficacy	(23 statements by 12 parents)
Guided navigation (n = 55 statements)	“*I’m gonna try that.*”—8-year-old girl with leukemia “*How? Oh, home.*”—6-year-old boy with leukemia	Guided navigation (n = 10 statements)	“*Just touch on the body where it’s bothering you.*”—mother of 8-year-old girl with leukemia. “*We go back to diary, there you go.*”—mother of 10-year-old boy with leukemia
Uncertainty (n = 6 statements)	“*Ahhh so what do I do now?*”—12-year-old boy with leukemia. “*Whoops. Is this…it works! How do I undo it?*”—10-year-old boy with lymphoma “*Need some help.*”—6-year-old boy with leukemia	Uncertainty (n = 6 statements)	“*Yeah little scroll bar can you see how now it’s not but maybe it’s just a little big of lag or something*”—father of 6-year-old girl with leukemia “*There’s characters I would’ve totally missed the characters*”—mother of 6-year-old boy with leukemia “*For the trends uhhh oh sorry that was not it that was right there and we go back and we help I hit it again*”—mother of 9-year-old girl with leukemia
Self-navigation (n = 15 statements)	“*Alright I gotta get my typing hands for this.*”—10-year-old boy with leukemia “*There see I clicked save.*”—9-year-old girl with leukemia “*Boop. I’m going to type a letter.*”—6-year-old boy with leukemia	Perception of the child’s self-efficacy (n = 7 statements)	“*… a lot of the times when he is in school, he does sight words right so after he sees some of those words, he’ll put together that you know when he does the answer, he can figure out which one to pick out after that so first time maybe*”—mother of 6-year-old boy with leukemia “*It’s very uh, user friendly, especially for young kids, um I do like that.*”—mother of 11-year-old girl with leukemia
Computer playfulness	(63 statements by 10 children)	Computer playfulness	(29 statements by 9 parents)
Interaction (n = 22 statements)	“*That’s cool they get little icons for them I like that that’s really nice to have.*”—12-year-old boy with leukemia “*Oh, I like the fishies.*”—6-year-old boy with leukemia “*I want to him to have hair. That one.*”—10-year-old boy with lymphoma	Interaction (n = 10 statements)	“*Let me see. Oh, that is so you. That’s so cute.*”—mother of 8-year-old girl with leukemia “*Making it have a redhead? Haha ok*”, mother of 6 years boy with leukemia “*Mohawk!*” mother of 10-year-old boy with leukemia
Suggestions (n = 22 statements)	“*The only thing I would change is make him [the avatar] have like movement almost instead of looking like a statue.*”—12-year-old boy with leukemia “*To make it better you need be able to change the fish and feed it.*”—10-year-old boy with leukemia “*Maybe allow the chicken to grow…So every time you’ve come back from the checkup, the chicken has grown.*”—6-year-old boy with leukemia “*Taking care of the person…giving them a Band-Aid.*”—6-year-old girl with leukemia	Suggestions (n = 10 statements)	“*More options and you know, I like these little things on the home screen that, you know, you don’t necessarily think, like the water, like the flowerpots. You don’t necessarily think they can do anything and like he was trying to touch the fish, like, seeing, you know, what could pop up by touching. You know, if you touch on the fish, is there something like you could feed your fish or you know, just things like that, I think would be fun. I’m trying to think of what. Like that age of kids, what they would like.*”—mother of 10-year-old boy with lymphoma “*Yeah I like the rewards idea that you mentioned that they could earn she would be able to earn points and then I could say if she’s not wanting to brush her teeth then I could say hey you can some earn some points in your app and then you could use that to unlock something or whatever the rewards would be*…”—father of 6-year-old girl with leukemia
General endorsement (n = 10 statements)	“*This is silly.*”—8-year-old girl with leukemia “*I’m also gonna add just a little more.*”—9-year-old girl with leukemia	General endorsement (n = 9 statements)	“*Very customizable that’s pretty cool*”—mother of 12-year-old boy with leukemia “*I like the cute daily missions to motivate them to do stuff so they can buy things.*”—mother of 6-year-old boy with leukemia “… *to make it personal*”—mother of 8-year-old girl with leukemia
Self-representation (n = 9 statements)	“*Oh see that yep that definitely looks like me but I’ve got a little goldish hair there we go.*”—10-year-old boy with leukemia “*Yeah, that’s my hair.*”—6-year-old boy with leukemia		
Perceived enjoyment	(26 statements by 11 children)	Perceived enjoyment	(17 statements by 8 parents)
Specific feature endorsement (n = 17 statements)	“*I can like write about my day in the diary and I can edit my own character and uh ummm and I think that’s really it.*”—9-year-old girl with leukemia “*And then you can draw, you can see what you paint.*”—9-year-old boy with leukemia “*I like this because it has drawing.*”—10-year-old boy with leukemia	Specific feature endorsement (n = 10 statements)	“*I like how they can customize their avatar.*”—mother of 6-year-old boy with leukemia “[my child] *likes to draw so. Very cool*”—mother of 11-year-old girl with leukemia
General endorsement (n = 9 statements)	“*Nothing needs to be added this is perfect how it is.*”—10-year-old boy with leukemia “*I like all of it.*”—6-year-old boy with leukemia	General endorsement (n = 7 statements)	“*I think it’s great. I have nothing bad to say about it.*”—mother of a 12-year-old boy with leukemia “*Yeah I think that looks good*”—father of 9-year-old boy with leukemia
**Perceived usefulness**			
Job relevance	(52 statements by 12 children)	Job Relevance	(69 statements by 14 parents)
Personal relevance (n = 20 statements)	“*The bruises actually happen, and they go all the way up my arm.*”—8-year-old boy with leukemia “*I want him to look like me.*”—10-year-old boy with leukemia “*Best thing about today is that I get unaccessed.*”—8-year-old girl with leukemia	Personal relevance (n = 21 statements	“*I think that is great. Just because, the days tend to, what’s the word I’m looking for, they all start mushing together when you’re in a stressful situation and, um, having the data on there would be really helpful. And then, you can write it down on a piece of paper, but I would lose the piece of paper, personally. So be able to log in and have access to all that information that came straight from the child, that’s huge*”—mother of 12-year-old boy with leukemia “*Mhm I think it has some really good features I mean I know they press this daily missions type of thing a lot when she first came in but they don’t I haven’t heard them talk about it as much like bathing each day and brushing teeth and stuff, but this would be a good place to reinforce that.*”—father of 6-year-old girl with leukemia
Functional capacity/application (n = 11 statements)	“*I think it’s a good way to make kids that are stressed out about having cancer this will like make it less scary for them.*”—12-year-old boy with leukemia. “*To show how I’m feeling and where I’m hurting.*”—8-year-old girl with leukemia	Functional capacity/application (n = 17 statements)	“*I like how you’re taking a record of how they’re feeling and letting them express the way they are feeling better. They can explain a little bit better and go into more detail.*”—father of 9-year-old boy with leukemia “*Then you can see how you’re feeling on different chemos too. That’s pretty awesome*”—mother of 10-year-old boy with leukemia
Reporting preferences (n = 21 statements)	“*Because writing on paper would be so hard and we don’t even have paper in the hospital.*”—10-year-old boy with leukemia “*It kinda just feels easier than writing it on paper because then my hand gets like really tired from writing fast.*”—9-year-old girl with leukemia “*I have a hard time explaining what’s wrong.*”—12-year-old boy with leukemia	Suggestions (n = 5 statements)	“*I think it’s really good. I guess it just depends on the age, if they have the attention span to go through all of the things. Like he went through the pain one, but did he go through all of those questions for fatigue? Or is the fatigue one pretty short?*”—mother of 8-year-old boy with leukemia “*Now this might be, maybe not with this app. But would there be any way to connect kids in similar situations that they could talk to each other?*”—mother of 11-year-old girl with leukemia “*I think if we were like in the hospital long-term, yes, it would be my preference. It probably would take a lot of time to do it if I was just coming like one day. It would probably be clearer just to get like an oral report of how he feels. But if he was in the hospital for multiple days and he could fill it out every morning after he brushes his teeth or whatever, I think that would be interesting.*”—mother of 6-year-old boy with leukemia
		Relatable technology (n = 3 statements)	“*It’s a better visual which I think sometimes is easier if the kids are, you know, gaming*”—mother of 10-year-old boy with leukemia “*The tablet, I think. Yeah. Maybe some of both. But mostly he just does his work on the tablet.*”—mother of 6-year-old boy with leukemia
		Developmental relevance (n = 23 statements)	“*I think he would definitely do the app better. He’s having some neuropathy and so it’s hard to hold a pencil, anyway.*”—mother of 8-year-old boy with leukemia “*She’ll think of things later on. She’s like, they said this so what does this mean or what if this happens and it’s like—okay. And I think if we had something like this that she could do that*….”—mother of 11-year-old girl with leukemia
Result demonstrability	(8 statements by 4 children)	Result demonstrability	(48 statements by 13 parents)
Functional capacity (n = 5 statements)	“*Poor person doesn’t get one day to feel good.*”—10-year-old boy with leukemia. “*On her neck that’s what it was sending how is her neck hurt.*”—10-year-old boy with leukemia “*I liked how it shows my own graph.*”—10-year-old old boy with lymphoma.	Functional capacity (n = 9 statements)	“*that’s kind of neat it uh helps guide and see where you know where you are you can see you can do this like this shows the week of the month*”—mother of 6-year-old boy with leukemia “*Um I like the simplicity of the menu … and all the data is really easy to find. I like the color scale to give it a nice little visual and the faces get sadder as you go so you know it’s more subdued*”—father of 6-year-old girl with leukemia
General endorsement (n = 3 statements)	“*That’s really cool.*”—12-year-old boy with leukemia. “That’s cool.”—8-year-old girl with leukemia.	General endorsement (n = 21 statements)	“*I like the history*”—mother of 8-year-old girl with leukemia “*Simple enough*”—father of 9-year-old boy with leukemia
		Interpretation (n = 11 statements)	“*She not only has a headache up here but down there is hurting too*”—mother of 10-year-old boy with leukemia “*Numb/tingly, yeah*. [seems to move to mouth]—*and then little sores and pain*”—mother of 10-year-old boy with lymphoma
		Suggestions for improvement (n = 7 statements)	“*The only thing I would suggest on here is like not so hard to read*”—mother of 10-year-old boy with leukemia “*That way you know you’re on the back instead of the front*”—father of 9-year-old boy with leukemia “*Maybe, I was touching the circle, not the foot so maybe that’s it*”—mother of 6-year-old boy with leukemia

## Data Availability

The data presented in this study are available on request from the corresponding author to external researchers working under an institution with a Federal Wide Assurance (FWA) who are willing to enter into formal research relationships to ensure that the data will be used for scientific purposes in the public interest, that patient privacy will be protected, and that all other risks to participants will be minimized.

## Supplementary Materials

The following supporting information can be downloaded at https://www.mdpi.com/article/10.3390/children11101215/s1: Table S1: Child usability results; Table S2: Parent usability results.
