# Supplementary material for: Usability Evaluation of the Revised Color Me Healthy Symptom Assessment App: Perspectives of Children and Parents
Source: Children (Basel). 2024 Oct 4;11(10):1215. doi: 10.3390/children11101215 (PMC11506821; doi:10.3390/children11101215)
Supplement: Supplementary file 1 [file children-11-01215-s001.zip › children-3173603-supplementary.pdf]

Supplementary Table S1: Child Usability Results

Task	Cycle 1 (n=5)			Cycle 2 (n=5)			Cycle 3 (n=4)		
	Completed Independently	Required Verbal Prompt	Required Physical Prompt	Completed Independently	Required Verbal Prompt	Required Physical Prompt	Completed Independently	Required Verbal Prompt	Required Physical Prompt
Pain as a general symptom									
Access checkup page	5	0	0	5	0	0	4	0	0
Indicate pain as a general symptom	5	0	0	4	1	0	4	0	0
Rate pain severity	5	0	0	4	1	0	4	0	0
Rate pain bother/distress	5	0	0	4	1	0	4	0	0
Localize pain to an area of the body									
Select an area of the body	2	3	0	4	1	0	4	0	0
Rate pain severity for that area	3	2	0	4	1	0	4	0	0
Rate pain bother/ distress for that area	4	1	0	4	1	0	4	0	0
Mark pain on the front-facing body part	4	1	0	4	1	0	3	1	0
Select the reverse side of the body part	3	1	0	4	1	0	4	0	0
Mark pain on the rear-facing body part	3	1	0	4	1	0	4	0	0
Return to home screen	3	2	0	5	0	0	4	0	0
Review pain reports/entries									
Access history page	4	0	1	4	0	0	4	0	0
Locate the general pain report	3	1	1	4	0	0	4	0	0
Locate the severity and distress ratings	3	2	0	4	0	0	4	0	0
Locate the localized pain report	2	2	1	4	0	0	4	0	0
Locate the severity and distress ratings	3	1	1	4	0	0	4	0	0

Supplementary Table S2: Parent Usability Results

Task	Cycle 1 (n=5)			Cycle 2 (n=5)			Cycle 3 (n=4)		
	Completed independently	Required Verbal Prompt	Required Physical Prompt	Completed Independently	Required Verbal Prompt	Required Physical Prompt	Completed Independently	Required Verbal Prompt	Required Physical Prompt
Access history feature	5	0	0	5	0	0	4	0	0
Daily pain report									
Locate pain on the daily general symptom report	5	0	0	5	0	0	4	0	0
Locate the severity and distress rating	5	0	0	5	0	0	4	0	0
Locate a report of pain localized to an area of the body	4	1	0	5	0	0	4	0	0
Locate the severity and distress ratings	5	0	0	5	0	0	4	0	0
Longitudinal pain reports									
Access longitudinal symptom reporting feature	5	0	0	5	0	0	4	0	0
Select and review longitudinal pain-related data based on a one-week interval	4	1	0	5	0	0	4	0	0
Select and review longitudinal pain-related data based on a one-month interval	5	0	0	5	0	0	4	0	0
